# Comparison of Lactate Clearance with Established Risk Assessment Tools in Predicting Outcomes in Acute Upper Gastrointestinal Bleeding

**DOI:** 10.3390/jcm12072716

**Published:** 2023-04-05

**Authors:** Gabriel Allo, Johannes Gillessen, Dilan Gülcicegi, Philipp Kasper, Seung-Hun Chon, Tobias Goeser, Martin Bürger

**Affiliations:** 1 Department of Gastroenterology and Hepatology, Faculty of Medicine and University Hospital Cologne, University of Cologne, 50937 Cologne, Germany; 2 Department of General, Visceral and Cancer and Transplant Surgery, University Hospital of Cologne, University of Cologne, 50937 Cologne, Germany

**Keywords:** gastrointestinal bleeding, endoscopy, emergency medicine

## Abstract

Early risk stratification is mandatory in acute upper gastrointestinal bleeding (AUGIB) to guide optimal treatment. Numerous risk scores were introduced, but lack of practicability led to limited use in daily clinical practice. Lactate clearance is an established risk assessment tool in a variety of diseases, such as trauma and sepsis. Therefore, this study compares the predictive ability of pre-endoscopic lactate clearance and established risk scores in patients with AUGIB at the University Hospital of Cologne. Active bleeding was detected in 27 (25.2%) patients, and hemostatic intervention was performed in 35 (32.7%). In total, 16 patients (15%) experienced rebleeding and 12 (11.2%) died. Initially, lactate levels were elevated in 64 cases (59.8%), and the median lactate clearance was 18.7% (2.7–48.2%). Regarding the need for endoscopic intervention, the predictive ability of Glasgow Blatchford Score, pre-endoscopic Rockall score, initial lactate and lactate clearance did not differ significantly, and their area under the receiver operating characteristic curves were 0.658 (0.560–0.747), 0.572 (0.473–0.667), 0.572 (0.473–0.667) and 0.583 (0.483–0.677), respectively. Similar results were observed in relation to rebleeding and mortality. In conclusion, lactate clearance had comparable predictive ability compared to established risk scores. Further prospective research is necessary to clarify the potential role of lactate clearance as a reliable risk assessment tool in AUGIB.

## 1. Introduction

Despite technological innovations and significant improvements in the clinical management of patients with acute upper gastrointestinal bleeding (AUGIB), morbidity and mortality rates remain high [1,2].

Current international guidelines recommend early risk stratification in AUGIB to allocate patients to the appropriate medical treatment, which may improve outcomes and save resources [3,4]. Numerous assessment tools have been developed in recent years to stratify patients according to their individual risk of unfavorable outcomes. In clinical practice, the Rockall score and the Glasgow Blatchford score (GBS) are the most frequently used risk scores [5,6]. The Rockall score was derived to predict mortality, and the GBS predicts need for intervention in patients with AUGIB. However, despite their proven benefit [7], these risk scores are infrequently used, mainly due to lack of practicability [8]. Therefore, the development of an easy-to-calculate risk score that is able to accurately predict need for intervention and to assess mortality risk is warranted.

Lactate levels and lactate clearance are established prognostic markers in a variety of emergency and intensive care conditions, such as trauma and sepsis [9,10]. As a product of anaerobic glycolysis, lactate occurs in hypoxic tissue during severe sepsis or hemorrhagic shock. Recently, studies have shown an association of elevated lactate levels with unfavorable outcomes in AUGIB [9,11,12,13]. Additionally, lactate levels had similar prognostic abilities compared to established risk scores [14], and it was even shown that incorporating lactate into established risk assessment tools improves the risk scores’ predictive ability [13,14].

Lactate clearance may be a valuable tool to predict the need for hemostatic intervention in AUGIB. Ongoing bleeding might result in continuously increased lactate production, and thereby, a reduced lactate clearance might indicate need for intervention for this persistent bleeding source. However, the level of evidence regarding lactate clearance in the setting of AUGIB is quite limited. One study indicated that an impaired lactate clearance is associated with active bleeding, while another recent study found an association of the 3 h lactate clearance with mortality in patients with AUGIB [15,16]. However, both studies did not evaluate the predictive ability of pre-endoscopic lactate clearance to derive a need for endoscopic intervention.

Thus, we conducted this study to evaluate the value of lactate clearance as a pre-endoscopic risk assessment tool in AUGIB and to compare its predictive accuracy with established risk scores.

## 2. Materials and Methods

All patients who received an esophagogastroduodenoscopy (EGD) at the University Hospital Cologne between 1 January 2015 and 31 December 2019 were retrospectively identified from our endoscopy database. Inclusion criteria were as follows: age ≥ 18 years, initial presentation with signs and symptoms of AUGIB at our emergency department (ED), endoscopic assessment with an EGD, and lactate measurement at presentation at the ED as well as a subsequent measurement within six hours and before index-EGD. Cases were excluded if initial or subsequent lactate measurements were missing, as well as initial presentation at another hospital. Patients with variceal bleeding were also excluded since such risk scoring systems as GBS as well as pre-endoscopic (pRS) and full Rockall score (fRS) are not validated for patients with variceal bleeding. Moreover, hepatic dysfunction is associated with elevated lactate levels, and severe liver disease impairs lactate clearance in septic patients without translating to mortality difference [17,18].

The following information was retrieved from medical records of included patients: gender, age, time of presentation to the emergency department, systolic and diastolic blood pressure, heart rate, signs and symptoms of AUGIB (hematemesis, melaena, and hematochezia), comorbidities in accordance with the Charlson comorbidity index (CCI) [19], use of antithrombotic agents or anticoagulants, laboratory values at presentation (lactate, hemoglobin, and urea), subsequent lactate (defined as latest lactate value within six hours and before index endoscopy), endoscopic findings, endoscopic, radiological and surgical interventions for hemostasis, number of blood transfusions, date of rebleeding and death.

Lactate clearance was defined as (initial lactate-subsequent lactate)/initial lactate. Established risk assessment tools, such as GBS, pRS and fRS, were calculated as previously described [5,6].

Rebleeding was defined as endoscopic signs of AUGIB within 30 days after initial hemostasis was obtained during index endoscopy. Signs of shock were defined as the presence of hypotension (systolic blood pressure below 100 mmHg) and/or tachycardia (heart frequency > 100/min). The primary composite endpoint was the need for hemostatic intervention (endoscopic, radiological or surgical intervention). The 30-day mortality and rebleeding rate were considered secondary endpoints.

Continuous variables were expressed as means ± standard deviation and compared using Student’s t test. Categorical variables were presented as absolute and relative frequencies and analyzed by χ^2^ test. Receiver operating characteristic (ROC) curves were created and the area under the ROC curve (AUROC) with 95% confidence intervals was calculated. The Delong test was used to compare AUROCs for equality [20]. Additionally, the Youden Index was used to identify the cutoff score with the highest sum of specificity and sensitivity for each risk assessment tool. Univariate and multivariate analyses using the logistic regression model were used to identify the variables associated with the need for hemostatic intervention. Variables were included in the multivariate analysis if their *p*-value was <0.1 in the univariate analysis. The significance level of <0.05 was determined as statistically significant. Descriptive analysis was conducted using Statistical Package for the Social Sciences, version 28 (IBM, Armonk, NY, USA) and Medcalc (MedCalc Software, Ostend, Belgium).

## 3. Results

### 3.1. Baseline Characteristics and Outcomes

Out of the 24.998 EGDs performed between 2015 and 2019, a sum of 2715 was performed due to suspected upper gastrointestinal bleeding. A total of 390 EGDs were performed due to suspected AUGIB in the ED; however, a summation of 107 cases met our strict inclusion criteria and were included in the final analysis. Baseline characteristics of the study population are described below in Table 1. The median age of included patients was 70 years, and of those patients 48 (44.9%) were women. The median CCI was six and antithrombotic agents or anticoagulants were taken by 34 (32.1%) and 26 (24.8%) of the patients, respectively. A total of 46 (43.0%) presented with signs of shock, and melaena was the most frequent clinical sign of bleeding (66.4%). Active bleeding was detected in 27 (25.2%) cases, and peptic ulcers were the most common bleeding etiology in 40 (38.1%) of the cases. In 64 (59.8%) patients, initial lactate was elevated; the median lactate clearance was 18.7%, the median GBS was 12 (IQR 9–14), the median pRS was 4 (IQR 3–5) and the median fRS was 6 (IQR 4–7). In total, 63 (60%) patients received a median of 2 (IQR 0–4) blood transfusions. Endoscopic, radiological and/or surgical hemostatic intervention was determined as necessary in 35 patients (32.7%), and 16 (15.0%) experienced rebleeding. In total, 12 of 107 (11.2%) patients died within 30 days after presentation. See Table 2 for clinical outcomes.

### 3.2. Performance of Risk Assessment Tools in Predicting Intervention, Mortality and Rebleeding

**Figure 1:**
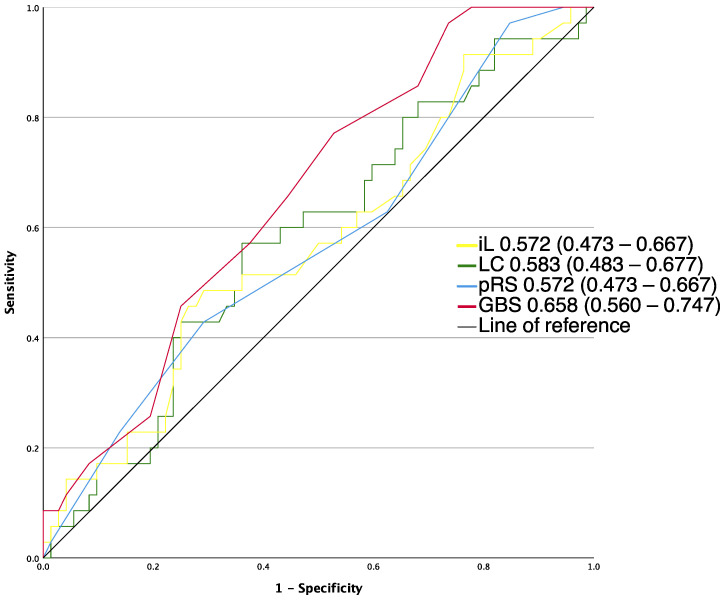
AUROCs of risk assessment tools for the prediction of need for intervention, not applicable to full Rockall Score. GBS, Glasgow–Blatchford score; iL, initial lactate; LC, lactate clearance; p-RS, pre-endoscopic RS.

**Figure 2:**
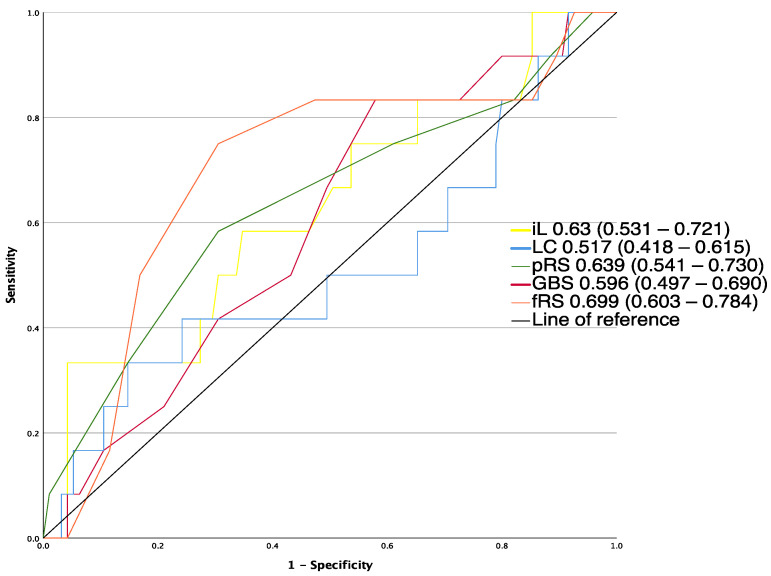
AUROCs of risk assessment tools for the prediction of 30-day mortality fRS, full Rockall score; GBS, Glasgow–Blatchford score; iL, initial lactate; LC, lactate clearance; p-RS, pre-endoscopic RS.

**Figure 3:**
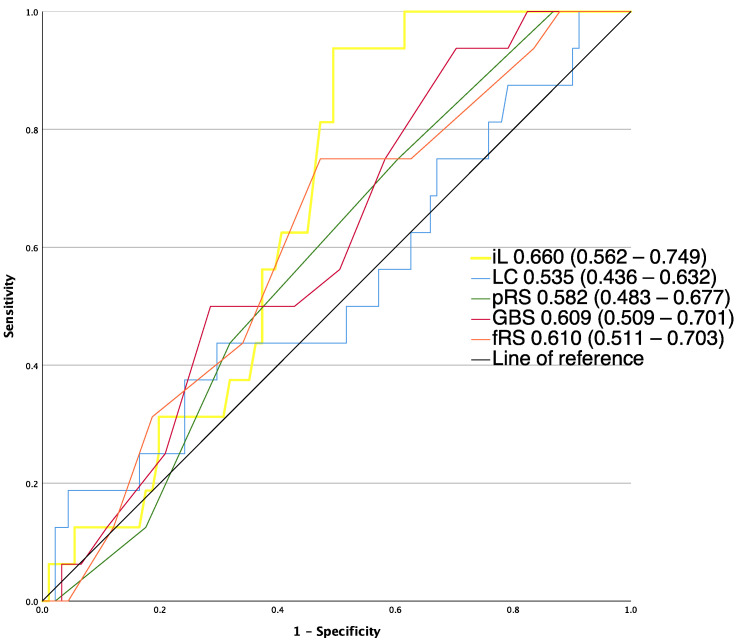
AUROCs of risk assessment tools for the prediction of and rebleeding fRS, full Rockall score; GBS, Glasgow–Blatchford score; iL, initial lactate; LC, lactate clearance; p-RS, pre-endoscopic RS.

AUROCs for the risk assessment tools for the analyzed outcomes are described in Figure 1, Figure 2 and Figure 3. Of all pre-endoscopic risk assessment tools, GBS (0.658 (0.560–0.747)) had the highest AUROC for predicting the need for hemostatic intervention, but the difference of discriminative ability did not reach any statistical significance (*p* > 0.05) compared to the lactate clearance (0.583 (0.483–0.677), pRS (0.572 (0.473–0.667)) and initial lactate (0.572 (0.473–0.667)).

When focusing on the composite endpoint of any intervention or death (including red blood cell transfusion, endoscopic treatment, interventional radiology, surgery or death), as described in a recent large multicenter study [21], GBS (0.791 (0.702–0.864)), pRS (0.713 (0.618–0.797)) and initial lactate (0.646 (0.548–0.736)) performed superior to lactate clearance (0.501 (0.403–0.6)), and GBS also outperformed the initial lactate. The difference of AUROCs of GBS and pRS was statistically non-significant (*p* > 0.05)

A total of 12 patients (11.2%) died within 30 days after presentation. fRS had the highest AUROC for predicting mortality (0.699 (0.603–0.784)), but the difference of discriminative ability did not reach statistical significance (*p* > 0.05) compared to pRS (0.639 (0.541–0.730)), initial lactate (0.63 (0.531–0.721)), GBS (0.596 (0.497–0.69)) and lactate clearance (0.517 (0.418–0.615)).

Rebleeding was observed in 16 of all 107 patients (15.0%). Initial lactate had the highest AUROC for predicting rebleeding (0.66 (0.562–0.749)), but the difference of discriminative ability did not reach statistical significance (*p* > 0.05) compared to fRS (0.61 (0.511–0.703)), GBS (0.609 (0.509–0.701)), pRS (0.582 (0.483–0.677)) and lactate clearance (0.535 (0.436–0.632)).

The overall performance of all analyzed risk assessment tools in predicting intervention, rebleeding as well as 30-day mortality was poor, indicated by AUROC values lower than 0.7.

### 3.3. Performance of Bleeding Risk Scoring Systems at Optimal Cutoffs

The cutoffs with the highest sum of sensitivity and specificity for predicting hemostatic intervention were ≤4 for pRS, and ≤10 for the GBS, ≤23.8% for lactate clearance and ≤3.8 mmol/L for initial lactate (see also Table 3).

Forty-two patients (39.3%) had a GBS of ≤10 and were classified as low risk; eight (19%) received hemostatic intervention. The sensitivity, specificity, positive predictive value and negative predictive value for this cut off was 22.9%, 52.8%, 41.5%, 81.0%. Four patients (9.5%) experienced rebleeding and two (4.8%) died.

Seventy-one patients (66.4%) had a pRS of ≤4 and were classified as low risk; twenty (28.2%) received hemostatic intervention. In this case, the sensitivity, specificity, positive predictive value and negative predictive value for this cut off were 42.9%, 70.8%, 41.7%, and 71.8%. Nine patients (12.7%) in this group experienced rebleeding, and five patients (7.0%) died.

Sixty patients (56.1%) had a lactate clearance of ≤23.8% and were classified as low risk; fifteen (25.0%) received hemostatic intervention. The sensitivity, specificity, positive predictive value and negative predictive value for this cut off were 57.1%, 63.9%, 42.6%, and 75%. Nine patients (15.0%) in this group experienced rebleeding, and seven patients (11.7%) died.

Sixty-eight patients (64.5%) had an initial lactate level of ≤3.8 mmol/l and were classified as low risk; eighteen (26.1%) received hemostatic intervention. The sensitivity, specificity, positive predictive value and negative predictive value for this cut off were 48.6%, 70.8%, 44.7%, and 73.9%. Ten (14.5%) patients in this group experienced rebleeding, and six patients (8.7%) died.

### 3.4. Factors Associated with the Need for Hemostatic Intervention

Univariate analysis identified age, urea and metastatic solid tumor as significant factors associated with the need for intervention. In multivariate analysis, urea and metastatic solid tumor were significantly associated with the need for intervention (see Table 4).

## 4. Discussion

In this study, we demonstrate that lactate clearance is not inferior to established risk assessment tools in predicting need for hemostatic intervention, rebleeding or death in patients with AUGIB. However, the predictive accuracy of all analyzed pre-endoscopic risk assessment tools was poor (AUROC < 0.7) regarding these endpoints.

Early risk assessment in the setting of AUGIB is imperative and recommended by international guidelines to allocate patients to the appropriate medical treatment, especially regarding timing of endoscopy and possible outpatient management, which may improve outcomes as well as save costs and resources. A variety of risk scores have been validated; however, according to a national survey, only half of gastroenterologists use them due to lack of utility. Most risk assessment tools include a multitude of variables and appear impractical. Therefore, the serial measurement of a single, frequently used laboratory parameter for appropriate risk stratification in AUGIB may garner the interest of medical practitioners.

The ability of lactate clearance to predict important outcomes in AUGIB has not been extensively examined until now. We chose to include cases with subsequent lactate measurements within six hours, reflecting the current recommendations of guidelines on sepsis management, which suggest a serial lactate measurement especially in the first six hours to guide therapy [22]. These recommendations are based on numerous studies indicating that the 6 h-lactate clearance is a valuable predictor of survival [22,23]. Current endoscopy guidelines recommend performing endoscopy within the first 24 h after AUGIB. In a recent randomized controlled study by Lau et al., survival did not differ between high-risk patients undergoing urgent (<12 h) vs. early (within 12–24 h) endoscopy [24]. Moreover, there was a trend toward more active bleedings and interventions performed in the urgent group without improving survival. Additionally, the results of a recent large territory-wide observational study indicate that urgent endoscopy within six hours might be associated with worse outcomes underlining the importance of prior resuscitation and hemodynamic stabilization before rushed endoscopy [25]. Lactate clearance might in turn be a valuable tool to guide the resuscitation and timing of endoscopy. In their retrospective study, T. Wada et al. showed that lactate clearance was associated with active bleeding in critically ill patients with AUGIB [15]. In their study, all patients received EGD within six hours, but information on the percentage of subsequent lactate levels, that were measured after hemostatic procedures were performed, was lacking. The measured lactate clearance might have been significantly impacted by prior hemostatic procedures performed between initial and subsequent lactate measurements. Therefore, the results from this study should be interpreted with caution. In our study, we chose to include only patients with two subsequent lactate measurements before endoscopy to describe the authentic lactate clearance’s ability to serve as a pre-endoscopic risk assessment tool. We excluded cases with subsequent lactate measurements after more than six hours since this time span appeared impractical for early pre-endoscopic risk assessment. In a recent study, the 3 h lactate clearance was significantly associated with in-hospital mortality (AUROC 0.756) in the context of AUGIB [16]. In our study, the median time between subsequent lactate measurements was comparable with 2.9 h (IQR 1.7–4.5 h), but the aforementioned performance of lactate clearance to predict mortality appeared more impressive than in our study. However, the authors did not give any information about the timing of endoscopy or hemostatic procedures between measurements, complicating the comparability of our results.

It has been hypothesized that an insufficient lactate clearance is associated with active bleeding. Since lactate is a product of anerobic glycolysis occurring in hypoxic tissues, an ongoing bleeding could result in continuously increased lactate production and thus, a reduced lactate clearance might indicate the need for intervention of a persistently bleeding source.

Interestingly, in our study, lactate clearance had poor predictive ability, rejecting the initial hypothesis. It has been shown before that persistent elevated lactate levels during sepsis are partly due to altered lactate utilization and not a result of lactate overproduction occurring in hypoxic tissue [26]. Furthermore, evidence indicates that lactate production may actually be secondary to adrenergic stimulation [27,28]. An increased production of catecholamines might be the primary cause of elevated lactate levels, and thereby, the ability of lactate clearance to predict persistent bleeding and need for intervention might be severely impaired.

The need for endoscopic, radiological or surgical hemostatic intervention is one of the most relevant questions in the setting of AUGIB. Therefore, we chose the need for hemostatic intervention as the primary endpoint. The composite endpoint “intervention or death” used in the many previous studies [21,29] comprises the need for transfusion therapy. In patients with serious comorbidity, non-bleeding-related anemia and transfusion requirement frequently occur. Thereby, the composite endpoint will be met despite the absence of gastrointestinal bleeding. Moreover, the predictive ability of risk scores, which incorporate anemia as a high-impact variable (pRS and GBS), will be displayed as being incorrectly high if transfusion for non-bleeding-related anemia has been performed. On the other hand, patients with serious comorbidity achieve high scores, even in the absence of AUGIB, because of anemia and higher levels of urea. This might have impaired the performance of GBS and pRS in predicting need for hemostatic intervention in our analysis. The performance of GBS and pRS to predict need for hemostatic intervention in our study was low; however, it was comparable to a recent study of Jimenez-Rosales et al. [30]. Furthermore, the predictive ability regarding need for intervention and mortality was comparable to the performance described by some participating medical centers in the large multicenter study by Stanley et al. [21]. Here, AUROCs of GBS ranged between 0.67–0.79 and 0.59–0.76 regarding the need for intervention and mortality. Additionally, in our study, the AUROCs of GBS and pRS regarding mortality were higher than in the study by Stokbro et al. [14].

The ability of various risk scores to predict outcome has extensively been examined before. In a large prospective multicenter study by Stanley et al., GBS had the highest AUROC for the composite endpoint of hemostatic intervention, transfusion or death (0.86) and outperformed multiple other risk scores, such as Rockall score and AIMS65 [21]. In our study, GBS also had the highest AUROC and outperformed initial lactate and lactate clearance in predicting this composite endpoint but performed equally to pRS. These divergent results could partly be explained by the significantly higher percentage of patients with known malignancy included in our study population compared to the above-mentioned study (25.2% vs. 14%) and the identification of metastatic malignancy as an important risk factor for need of hemostatic intervention. Since only pRS includes metastatic malignancy in its comorbidity variable with the highest score, the predictive performance might differ between these study populations. Furthermore, the presence of serious comorbidity, such as metastatic cancer or severe renal disease, was more commonly present in our study population compared to previous studies [21,31,32]. These comorbidities are often associated with severe anemia, which is also a variable of the Rockall score and GBS. Compared to the mentioned studies, the median hemoglobin level was significantly lower in our study (8.4 mg/dL vs. 11.2 mg/dL). Since the detected anemia in severely ill patients is regularly not due to AUGIB, the performance of risk scores might be impacted significantly, explaining their poor performance in our study.

Bleeding from malignancies was detected in 75 (2.7%) of all EGDs performed due to suspected AUGIB at our institution during the study period, which is comparable with prior studies [21]. However, in our final study population, the proportion of bleeding malignancies detected was significantly higher. Interestingly, the performance of analyzed risk scores did not differ in a subgroup analysis between patients with bleeding malignancies and the non-malignant bleeding group. Surprisingly, lactate clearance performed significantly better in predicting mortality in the malignant bleeding group (see Supplementary Materials Table S2). Of course, these results must be interpreted with caution given the small sample size of patients with bleeding malignancies (n = 12). The poor performance of these risk scores warrants the development and evaluation of new risk assessment tools. Recently, two new risk scores (MAP(ASH) and ABC score) were therefore introduced. The MAP(ASH) is a simple-to-calculate risk score with comparable predictive ability to existing risk scores [33]. The recently introduced ABC score was derived from a large multicenter cohort and showed good performance for predicting mortality in both upper and lower gastrointestinal bleeding, thus outperforming other analyzed risk scores [32]. Both risk scores included the American Society of Anesthesiologists score, taking into account patients’ comorbidity and promise improved predictive ability, even in severely ill patients. Unfortunately, we were unable to analyze these scoring systems, since albumin, which is an included variable in both scores, is not measured routinely in our ED. Further studies are needed to evaluate these scores in different study populations.

The addition of lactate to established scoring systems improved their predictive power [13,14], although, in our study, the addition of lactate clearance to GBS and pRS with different weighting did not improve the performance of scoring systems in predicting the need for hemostatic intervention (see Supplementary Materials Table S3).

The major limitations of this study stem from its retrospective and monocentric design; therefore, generalizability might be limited. We were unable to compare our findings with other validated risk scores, such as AIMS65 (albumin level < 30 g/dL, international normalized ratio > 1.5, altered mental status, systolic blood pressure ≤ 90 mm Hg, and age > 65 years) as well as the newly introduced ABC score and MAP(ASH) score because of missing data, to compute these risk scores. Mainly, albumin was missing since it is not routinely measured in our ED. Unfortunately, detailed information on transfusion management was not available in a significant proportion of the study population. Therefore, we cannot rule out differences in resuscitation management. However, resuscitation is performed in accordance with current guidelines at our hospital as a standard of care. Thus, it may be assumed that the results were not significantly impacted by differing resuscitation management.

Additionally, only a small number of patients who presented with AUGIB underwent serial lactate measurements within six hours and before EGD, and this might have resulted in selection bias. Patients who appeared unstable or critically ill are likely to be monitored more closely and receive more extensive serial blood testing. On the other hand, critically ill patients are more likely to receive urgent EGD before subsequent lactate measurements are performed. Our study population appears more critically ill compared to prior studies regarding hemoglobin levels and comorbidities, and therefore, our results might not be transferable to AUGIB patients in general.

However, we believe that applying our strict inclusion criteria ensured an undistorted evaluation of the predictive ability of lactate clearance in patients with AUGIB. Our results should be validated in a prospective trial to prevent selection bias.

## 5. Conclusions

In conclusion, lactate clearance was not inferior to established risk assessment tools in predicting relevant outcomes in AUGIB, although the overall performance of analyzed risk assessment tools was poor. New tools are required to accurately predict outcomes in patients with AUGIB.

## Figures and Tables

**Table 1:** Patient baseline characteristics.

	Total	Non-Intervention	Intervention	*p*-Value
Age (median (IQR))	70 (56–77)	68 (51–76)	73 (61–81)	0.027
Women, n (%)	48 (44.9)	32 (44.4)	16 (45.7)	0.901
Coronary heart disease	13 (12.1)	10 (13.9)	3 (8.6)	0.43
Congestive heart failure	15 (14.0)	8 (11.1)	7 (20.0)	0.214
Cerebrovascular disease	18 (16.8)	9 (12.5)	9 (25.7)	0.086
Peripheral vascular disease	19 (17.7)	11 (15.3)	8 (22.9)	0.336
Chronic pulmonary disease	12 (11.2)	11 (15.3)	1 (2.9)	0.056
Peptic ulcer disease	27 (25.2)	20 (27.8)	7 (20.0)	0.385
Diabetes without end-organ damage	23 (21.5)	13 (12.1)	10 (9.3)	0.214
Diabetes with end-organ damage	6 (5.6)	4 (5.6)	2 (5.7)	0.973
Moderate/severe renal disease	25 (23.4)	20 (27.8)	5 (14.3)	0.122
Non-metastatic tumor	14 (13.1)	8 (11.1)	6 (17.1)	0.385
Moderate/severe liver disease	20 (18.7)	15 (20.8)	5 (14.3)	0.415
Metastatic solid tumor	13 (12.1)	5 (6.9)	8 (22.9)	0.018
CCI median, (IQR)	6 (4–8)	6 (3–8)	7 (4–8)	0.09
Antithrombotic drug, n(%)	34 (32.1)	27 (37.5)	7 (20.6)	0.082
Anticoagulants, n (%)	26 (24.8)	14 (19.7)	12 (35.3)	0.084
Signs of bleeding, n (%)				
Hematemesis	39 (36.4)	24 (33.3)	15 (42.9)	0.922
Melaena	71 (66.4)	48 (66.7)	23 (65.7)	0.922
Hematochezia	13 (12.1)	9 (12.5)	4 (11.4)	0.874
Syncope	12 (11.2)	9 (12.5)	3 (8.6)	0.546
Pulse rate, median (IQR), beats per minute	83 (75–102)	82 (76–102)	86 (71–102)	0.718
Tachycardia	30 (28.0)	20 (27.8)	10 (28.6)	0.932
Systolic blood pressure, median (IQR), mmHg	120 (95–138)	125 (96–140)	117 (93–130)	0.16
Signs of shock	46 (43.0)	30 (41.7)	16 (45.7)	0.692
Bleeding etiologies, n (%)				
Peptic ulcers	40 (38.1)	21 (29.2)	19 (54.2)	0.012
Neoplasms	12 (11.4)	4 (5.6)	8 (22.9)	0.008
Others	41 (39.1)	33 (45.8)	8 (22.9)	0.022
Laboratory parameters, median (IQR)				
Urea (mg/dL)	79 (47–120)	76 (41–108)	88 (61–185)	0.023
Hemoglobin (g/dL)	8.4 (6.7–9.9)	8.6 (6.7–10.1)	7.9 (6.1–8.8)	0.057
Initial Lactate (mmol/L)	2.7 (1.6–5.4)	2.7 (1.6–5.0)	3.1 (1.7–6.0)	0.195
Subsequent Lactate (mmol/L)	1.8 (1.2–3.9)	2.0 (1.1–3.5)	1.6 (1.4–4.0)	0.87
Hours between lactate measurement,	2.9 (1.7–4.5)	2.9 (1.6–4.4)	3.2 (1.8–4.9)	0.450
Lactate clearance (%)	18.7 (2.7–48.2)	16.8 (−0.6–41.2)	28.9 (8.7–51)	0.376
Initial lactate elevated (%)	64 (59.8)	42 (58.3)	22 (62.9)	0.654
Risk scores median, (IQR)				
GBS	12 (9–14)	11 (8–14)	13 (11–15)	0.004
p-RS	4 (3–5)	4 (3–5)	4 (3–5)	0.123
f-RS	6 (4–7)	5 (4–6)	7 (6–9)	<0.001

CCI = Charlson Comorbidity Index, f-RS = full Rockall score, GBS = Glasgow Blatchford Bleeding Score, IQR = interquartile range, p-RS = pre-endoscopic Rockall score, SD = Standard deviation.

**Table 2:** Outcomes (%).

Outcome	Number of Patients (%)
Active Bleeding at Endoscopy	27 (25.2)
Endoscopic intervention	30 (29.1)
Radiological intervention	6 (5.6)
Surgical intervention	2 (1.9)
Any interventions	35 (32.7)
Patients received transfusions	63 (60)
Number of packed red-blood-cells transfused (median (range))	2 (0–4)
30-day mortality	12 (11.2)
30-day rebleeding rate	16 (15.0)

**Table 3:** Sensitivity, specificity, PPV, NPV of analyzed pre-endoscopic risk assessment tools for predicting need for intervention, using the cutoff with the highest sum of sensitivity and specificity.

	Cutoff Value	Identified Patients	Need for Intervention	Sensitivity %	Specificity %	PPV %	NPV %
Initial lactate	≤3.8 mmol/L	68 (64.5%)	18 (26.1%)	48.6	70.8	44.7	73.9
Lactate clearance	≤23.8%	60 (56.1%)	15 (25.0%)	57.1	63.9	42.6	75.0
GBS	≤10	42 (39.3%)	8 (19%)	22.9	52.8	41.5	81.0
pRS	≤4	71 (66.4%)	20 (28.2%)	42.9	70.8	41.7	71.8

GBS = Glasgow–Blatchford score; PPV = positive predictive value; pRS = pre-endoscopic Rockall score; NPV = negative predictive value.

**Table 4:** Multivariate analysis of parameters associated with need for hemostatic intervention.

	Odds Ratio	Confidence Interval	*p*-Value
Age	1.031	0.993–1.071	
Cerebrovascular disease	1.763	0.468–6.646	0.402
Chronic pulmonary disease	0.184	0.018–1.89	0.154
Metastatic solid tumor	4.007	1.018–15.775	0.047
Antithrombotic drugs	0.361	0.115–1.138	0.082
Anticoagulants	0.855	0.267–2.735	0.792
Urea (mg/dL)	1.006	1.000–1.013	0.047
Hemoglobin (g/dL)	0.831	0.66–1.046	0.114

## Data Availability

The data sets used and/or analyzed during the current study are available from the corresponding author on reasonable request.

## Supplementary Materials

The following supporting information can be downloaded at: https://www.mdpi.com/article/10.3390/jcm12072716/s1, Table S1: Subgroup analysis on the risk scores’ performance in patients with bleeding from malignancies and the non-malignant bleeding group; Table S2: Subgroup analysis on the analyzed endpoints in patients with bleeding from malignancies (n = 12) and the non-malignant bleeding group; Table S3: Comparison of risk scores’ performance after including lactate clearance with different weighting
