# Supplementary material for: Comparison of Lactate Clearance with Established Risk Assessment Tools in Predicting Outcomes in Acute Upper Gastrointestinal Bleeding
Source: J Clin Med. 2023 Apr 5;12(7):2716. doi: 10.3390/jcm12072716 (PMC10095270; doi:10.3390/jcm12072716)
Supplement: Supplementary file 1 [file jcm-12-02716-s001.zip › jcm-2308988-supplementary.pdf]

Table S1. Subgroup analysis on the analyzed endpoints in patients with bleeding from malignancies (n=12) and the non-malignant bleeding group (percentages in columns).

	Bleeding malignancy	Non-malignant bleeding group	p-value
Hemostatic intervention	8 (66.7)	27 (28.4)	0.008
Mortality	2 (16.7)	10 (10.5)	0.525
Rebleeding	2 (16.7)	14 (14.7)	0.860

Table S2. Subgroup analysis on the risk scores' performance in patients with bleeding from malignancies (n=12) and the non-malignant bleeding group.

	Bleeding malignancy	Non-malignant bleeding group	p-value
Hemostatic intervention			
Lactate clearance	0.688	0.542	0.432
Initial lactate	0.406	0.593	0.300
pRS	0.500	0.572	0.717
GBS	0.734	0.653	0.671
Mortality			
Lactate clearance	0.850	0.458	0.012
Initial lactate	0.550	0.641	0.657
pRS	0.525	0.645	0.616
fRS	0.625	0.689	0.356
GBS	0.525	0.602	0.283
Rebleeding			
Lactate clearance	0.450	0.553	0.658
Initial lactate	0.500	0.675	0.316
pRS	0.525	0.583	0.744
fRS	0.425	0.628	0.353
GBS	0.525	0.616	0.732

fRS, full Rockall score; GBS, Glasgow-Blatchford score; p-RS, pre-endoscopic RS.

Table S3. Comparison of risk scores' performance after including lactate clearance with different weighting.

	Points	AUROCs	p-value
GBS	LC Cut-off >23.8%*		
	1	0.658vs0.673	0.127
	2	0.658vs0.685	0.149
	3	0.658vs0.692	0.190
	4	0.658vs0.699	0.215
	5	0.658vs0.703	0.245
	6	0.658vs0.704	0.285
pRS			
	1	0.582vs.0.576	0.821
	2	0.582vs.0.568	0.782
	3	0.582vs.0.557	0.709

AUROCs: Area under the receiver operating characteristic; GBS= Glasgow-Blatchford score; LC= lactate clearance; pRS= pre-endoscopic Rockall score.

*As identified by Youden-Index.
